# Effects of Meditation Training and Non-Native Language Training on Cognition in Older Adults: A Secondary Analysis of a Randomized Clinical Trial

**DOI:** 10.1001/jamanetworkopen.2023.17848

**Published:** 2023-07-14

**Authors:** Harriet Demnitz-King, Florence Requier, Tim Whitfield, Marco Schlosser, Julie Gonneaud, Caitlin Ware, Thorsten Barnhofer, Nina Coll-Padros, Sophie Dautricourt, Marion Delarue, Olga M. Klimecki, Léo Paly, Eric Salmon, Ann-Katrin Schild, Miranka Wirth, Eric Frison, Antoine Lutz, Gaël Chételat, Fabienne Collette, Natalie L. Marchant

**Affiliations:** 1 Division of Psychiatry, University College London, London, United Kingdom, W1T 7NF; 2 GIGA-CRC In Vivo Imaging, University of Liège, Liège, Belgium; 3 Psychology and Cognitive Neuroscience Unit, University of Liège, Liège, Belgium; 4 Department of Psychology, Faculty of Psychology and Educational Sciences, University of Geneva, Geneva, Switzerland; 5 Normandie Univ, UNICAEN, INSERM, U1237, Physiopathology and Imaging of Neurological Disorders (PhIND), Institut Blood and Brain @ Caen-Normandie, Cyceron, France; 6 Centre de recherches psychanalyse, médecine et société (CPRMS), Université de Paris, Paris, France; 7 School of Psychology, University of Surrey, Guildford, United Kingdom; 8 Alzheimer’s Disease and Other Cognitive Disorders Unit, Hospital Clinic, August Pi i Sunyer Biomedical Research Institute (IDIBAPS), Barcelona, Spain; 9 Neurology Department, University Hospital, Caen, France; 10 Clinical Psychology and Behavioral Neuroscience, Faculty of Psychology, Technische Universität Dresden, Dresden, Germany; 11 Department of Psychiatry, Medical Faculty, University of Cologne, Cologne, Germany; 12 German Center for Neurodegenerative Diseases (DZNE), Dresden, Germany; 13 Bordeaux Population Health Center, University of Bordeaux, INSERM, EUCLID/F-CRIN Clinical Trials Platform, CHU Bordeaux, Bordeaux, France; 14 Service d’information médicale, CHU Bordeaux, Bordeaux, France; 15 Eduwell team, Lyon Neuroscience Research Center INSERM U1028, CNRS UMR5292, Lyon 1 University, Bron, Lyon, France

## Abstract

**Importance:**

Nonpharmacological interventions are a potential strategy to maintain or promote cognitive functioning in older adults.

**Objective:**

To investigate the effects of 18 months’ meditation training and 18 months’ non-native language training on cognition in older adults.

**Design, Setting, and Participants:**

This study was a secondary analysis of the Age-Well trial, an 18-month, observer-masked, randomized clinical trial with 3 parallel arms. Eligible participants were community-dwelling adults aged 65 years and older residing in Caen, France. Participants were enrolled from November 24, 2016, to March 5, 2018, and randomly assigned (1:1:1) to meditation training, non-native language (English) training, or no intervention arms. Final follow-up was completed on February 6, 2020. Data were analyzed between December 2021 and November 2022.

**Interventions:**

The 18-month meditation and non-native language training interventions were structurally equivalent and included 2-hour weekly group sessions, daily home practice of 20 minutes or longer, and 1 day of more intensive home practice. The no intervention group was instructed not to change their habits and to continue living as usual.

**Main Outcomes and Measures:**

Cognition (a prespecified secondary outcome of the Age-Well trial) was assessed preintervention and postintervention via the Preclinical Alzheimer Cognitive Composite 5 (PACC5), and composites assessing episodic memory, executive function, and attention.

**Results:**

Among 137 randomized participants, 2 were excluded for not meeting eligibility criteria, leaving 135 (mean [SD] age, 69.3 [3.8] years; 83 female [61%]) eligible for analysis. One participant among the remaining 135 did not complete the trial. In adjusted mixed effects models, no interaction effects were observed between visit and group for PACC5 (F_2,131.39_ = 2.58; *P* = .08), episodic memory (F_2,131.60_ = 2.34; *P* = .10), executive function (F_2,131.26_ = 0.89; *P* = .41), or attention (F_2,131.20_ = 0.34; *P* = .79). Results remained substantively unchanged across sensitivity and exploratory analyses.

**Conclusions and Relevance:**

In this secondary analysis of an 18-month randomized trial, meditation and non-native language training did not confer salutary cognitive effects. Although further analyses are needed to explore the effects of these interventions on other relevant outcomes related to aging and well-being, these findings did not support the use of these interventions for enhancing cognition in cognitively healthy older adults.

**Trial Registration:**

ClinicalTrials.gov Identifier: NCT02977819

## Key Points

**Question:**

Does 18 months of either meditation training or non-native language training improve cognition in cognitively healthy older adults?

**Findings:**

In this secondary analysis of a randomized clinical trial that included 135 older adults, there was no evidence that meditation training or non-native language training significantly improved global cognition, episodic memory, executive function, or attention.

**Meaning:**

These findings suggest that neither meditation training nor non-native language training should be used as a method for improving cognition in cognitively healthy older adults.

## Teaser

This secondary analysis of a randomized clinical trial of older French adults examines whether training in meditation or non-native language improves scores for global cognition, episodic memory, executive function, or attention.

## Introduction

Aging is a risk factor for Alzheimer disease (AD), of which cognitive decline is a primary symptom. Despite ongoing pharmacological research, treatments to prevent or delay cognitive decline have yet to produce clinically meaningful results.^1,2^

Nonpharmacological interventions such as mindfulness-based interventions (MBIs; therapeutic approaches aimed at promoting attentional and emotional self-regulation, and fostering nonjudgemental awareness^3^) are increasingly being investigated for salutary cognitive effects. A meta-analysis of MBIs based on randomized clinical trials (RCTs) found small benefits on overall cognition (*k* = 15; *g* = 0.21; 95% CI, 0.04-0.38) and executive function (*k* = 8; *g* = 0.27; 95% CI, 0.05-0.50) in older adults.^4^ However, intervention lengths were relatively short (mean [SD], 7.7 [2.5] weeks). It is possible that interventions of longer durations could confer larger gains. Moreover, the studies’ methodological quality should also be considered, as only a minority had a low risk of bias.^4^ Proposed recommendations to improve methodological rigor include evaluating intervention adherence effects and comparing MBIs with both active and passive comparators.^4^ Recently, 2 methodologically rigorous RCTs (147 participants in an 8-week intervention^5^; 585 participants in an 18-month intervention^6^) examined the cognitive effects of MBIs relative to comparator interventions.^5,6^ While the MBIs conferred small improvements, cognitive gains of similar magnitudes were observed across comparator interventions.^5,6^ Neither study included a passive control, thus it is unclear whether MBIs confer cognitive benefits, or whether improvements reflect expectancy or practice effects.

Non-native language learning has also been posited as a promising intervention for enhancing cognition. However, research in older adults is sparse, with a systematic review identifying 9 studies, of which just 4 were RCTs.^7^ Positive effects on attentional switching, inhibition, and working memory were observed, albeit inconsistently.^7^ Inconclusive findings have been attributed to shortcomings in study designs (eg, intervention lengths) and methodological issues (eg, nonrandomized designs).^7,8^ However, 2 recent RCTs with longer intervention durations (16 and 30 weeks), which included non-native language training, active (brain training^9^ and strategy game^10^), and passive control groups, reported opposing findings (ie, positive effects on some cognitive domains vs no benefits).^9,10^ Additional RCTs are required to better understand the efficacy of non-native language learning in older adults.

Meditation and non-native language training have been theorized to affect cognition via distinct and partially overlapping mechanisms. For instance, both could enhance attention and executive function through increased cognitive control,^11,12^ and meditation training additionally through self-regulation.^11^ Furthermore, non-native language training has been postulated to enhance episodic memory by promoting more efficient cognitive processing (eg, semantic encoding),^13^ and meditation training through improved selective attention.^14^ Accordingly, both interventions may improve global cognition by enhancing cognitive processes across domains. Here, we conduct secondary analyses of the 18-month Age-Well RCT to investigate the cognitive effects of meditation and non-native language training.

## Methods

### Study Design

Age-Well was a monocentric, observer-masked, randomized clinical trial with 3 parallel arms: an 18-month meditation training intervention arm, an 18-month non-native language (English) training intervention arm, and an arm with no intervention. Participants completed a prescreening visit, with eligible participants invited to a baseline preintervention visit, before being randomized. A midintervention visit was performed after 9 months, and a postintervention visit performed at the end of the intervention. The preintervention and postintervention visits comprised multimodal assessments, including cognitive, behavioral, neuroimaging, and biological assessments, while the 9-month visit included select behavioral measures only. Age-Well received ethical approval from the Comité de Protection des Personnes Nord-Ouest III in Caen, and this study followed the Consolidated Standards of Reporting Trials (CONSORT) reporting guideline. Further details regarding the trial design, protocol, and statistical analysis plan, have previously been described^15,16^ and are available in Supplement 1.

### Participants

Participants were enrolled between November 24, 2016, and March 5, 2018. All participants were recruited from the general population, aged 65 years or older, native French speakers, retired for at least 1 year, received at least 7 years of education, had no evidence of major neurological or psychiatric disorders, and performed within normal ranges on standardized cognitive tests. Exclusion criteria included present or past regular or intensive meditation practice and speaking fluent English. Participants underwent a medical interview with a physician and completed a diagnostic test battery to verify criteria. eTables 1 and 2 in Supplement 2 contain additional information. All participants provided written informed consent before participation.

### Randomization and Masking

Following baseline visits, participants were randomized (1:1:1) to meditation training, non-native language training, or no intervention arms. Randomization was performed according to a randomization list with permuted blocks of varying size, which was generated centrally by a biostatistician. All study personnel were masked to treatment allocation. Only intervention facilitators, trial-independent statisticians, and data monitoring infrastructure staff were unmasked or partially unmasked.

### Interventions

The meditation and non-native language training interventions were structurally equivalent in overall course length, class time, and home activities, and matched in administration, dosage, duration, and level of expertise and number of facilitators per class. Interventions were manualized and comprised 2-hour weekly group sessions, daily home practice (minimum 20 minutes), and 1-day intensive practice (5 hours). Participants were encouraged to participate in all activities, and to not practice activities proposed in the other arms.

The meditation training consisted of an original secular program designed for Age-Well and based on preexisting interventions.^17,18,19^ The objective was to foster mindfulness, kindness, and compassion as additional psychological resources to support individuals to better cope with cognitive, physical, and psychological aspects of aging. It comprised 9 months of mindfulness meditation practice followed by 9 months of loving kindness and compassion meditation practice. Sessions included periods of group meditation (sitting or walking), sharing, and teaching.

The non-native language training was a cognitively stimulating program consisting of English exercises designed to reinforce participants’ abilities to understand, write, and speak English. Sessions were tailored to baseline English proficiency levels and incorporated oral comprehension and expression activities to facilitate acquisition of vocabulary and grammatical structures.

The no intervention group was requested not to change their habits and to continue living as usual. eAppendix 1 in Supplement 2 contains further intervention details.

### Measures

Cognitive composites possess higher sensitivity relative to individual neuropsychological tests, might be less susceptible to floor and ceiling effects, and reduce statistical multiplicity.^20^ We thus specified 1 global and 3 domain-specific composites at the preintervention and postintervention visits. Composites were created by computing the mean across scaled (*z*-transformed) cognitive test scores, with scores only calculable when data were available for all constituent measures. To facilitate interpretation, composite scores were restandardized using their baseline mean and standard deviation. Higher scores reflected better performance.

The Preclinical Alzheimer Cognitive Composite 5 (PACC5) is a validated global cognitive composite sensitive to detecting and tracking preclinical AD-related decline.^21^ It comprises 2 measures of episodic memory and 1 measure of executive function, semantic memory, and global cognition. In Age-Well, the Logical Memory test-story B (delayed recall), California Verbal Learning Test-II (CVLT-II; delayed free recall), Wechsler Adult Intelligence Scale (WAIS)-IV Coding (raw score), category fluency (total correct), and Mattis Dementia Rating Scale-2 (total score) were used. The episodic memory composite included 3 CVLT-II scores (sum of trials 1 through 5, immediate free recall, and delayed free recall), and 2 scores from the Logical Memory test-story B (immediate and delayed recall). The executive function composite comprised the Digit Span test backward (total correct), Trail-Making Test (TMT)-B (completion time), Stroop interference (completion time), and letter fluency (total correct). The attention composite comprised the Digit Span test forward (total correct), TMT-A (completion time), Stroop naming (completion time), and WAIS-IV Coding (raw score).

Baseline demographic and clinical characteristics were obtained from participants. Following the first intervention session, participants’ perceptions of intervention credibility and expectations of deriving benefit were measured via the Credibility/Expectancy Questionnaire.^22^ Intervention engagement was assessed via class attendance and total practice (ie, minutes spent in class and engaged in formal home practice). Furthermore, participants were classified as intervention responders or nonresponders based on a scale completed by facilitators assessing their perception of each participant’s intervention benefit. For the non-native language training group, improvement on an English language test was considered alongside facilitator ratings. Continuous measures of responsiveness were also created for both groups. eAppendix 2 in Supplement 2 contains further details.

### Sample Size

Age-Well was powered to detect an effect size of 0.75 for the trial’s coprimary outcomes (ie, volume and perfusion of the anterior cingulate cortex and insula), with 80% power and a 2-sided type I error of 1.25%.^16^ This resulted in a minimum of 126 participants (42 per arm), which was exceeded (137 total participants). Following guidance,^23^ post-hoc power analyses were not performed for this secondary outcome study.

### Statistical Analysis

**Figure 1.**
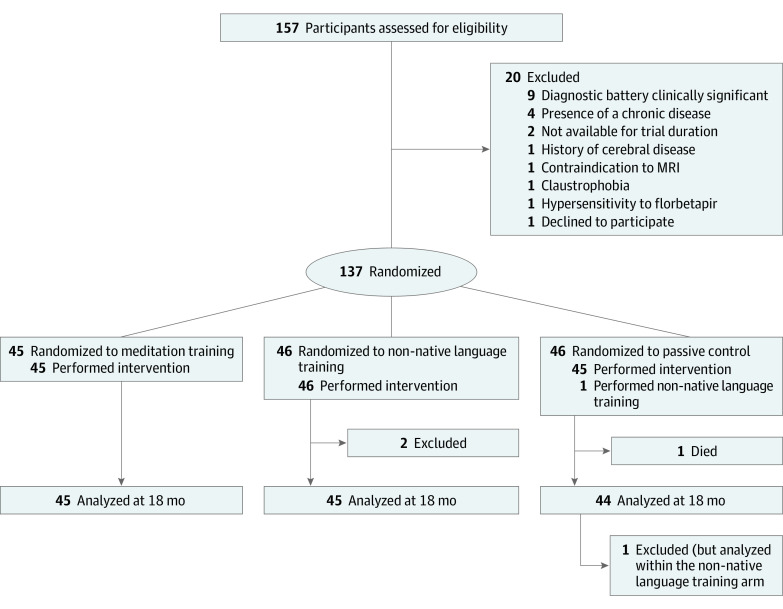
CONSORT Diagram of Study Enrollment and Randomization Of the 137 randomized participants, 2 were excluded by the trial steering committee from all secondary analyses for not meeting eligibility criteria (1 participant had history of head trauma [loss of consciousness for 1 hour or longer] and 1 participant received a clinical diagnosis of amyotrophic lateral sclerosis between the 9-month and postintervention visits, with a likely subclinical state at inclusion). Furthermore, 1 participant died during the follow-up (their data was included in mixed effects models for estimating intercepts only), and 1 participant revealed that they had not followed their allocated arm (randomized to no intervention but attended non-native language training and was analyzed within the non-native language training arm). CONSORT indicates Consolidated Standards of Reporting Trials; MRI, magnetic resonance imaging.

Statistical analysis followed a modified intention-to-treat strategy, with participants analyzed according to their allocated arm, except for participants excluded for not meeting major eligibility criteria as determined by the trial steering committee and 1 participant who deviated from their allocated intervention and was analyzed according to the intervention received (Figure 1).

Mixed effects models with participant-level random intercepts, estimated via restricted maximum likelihood, were fitted for each cognitive composite. Intervention effects were evaluated through the interaction between visit and trial arm. Post-hoc pairwise comparisons and within-arm changes were also examined. For all analyses baseline age, sex, and education level were included as fixed effects, with continuous variables mean-centered.

Amyloid deposition, a dementia risk marker known to affect cognition, was added as a fixed effect in a sensitivity analysis. Additional sensitivity analyses assessed intervention effects for participants who attended 20% or more of intervention classes (the a priori determined adequate minimum dose^15^) or were classified as responders.

Exploratory analyses further examined associations between participant characteristics and change in cognition. Adjusted linear regressions were conducted separately in the meditation and non-native language training arms, with change in composite scores as the outcome and the variable of interest (ie, age, sex, education, apolipoprotein ε4 status, amyloid deposition, vascular risk, credibility, expectancy, practice, responsiveness, and baseline composite scores) as the predictor. Variables were chosen based on association with dementia risk or potential to affect intervention engagement. In France, there are strict ethical restrictions about asking participants for their race and ethnicity. We did not receive ethical approval to do so, therefore we did not include race and ethnicity as a variable in this study.

Analyses were conducted in R version 4.0.2 (R Foundation for Statistical Computing), utilizing the *lme4* package for mixed effects models, *Anova* function for omnibus tests, and *emmeans* package for pairwise comparisons and estimated marginal means. Two-sided unadjusted and false discovery rate-adjusted (using the Benjamini and Hochberg method) *P*-values are reported, with *P* < .05 considered significant.

## Results

Of the 137 randomized participants, 135 were analyzed (mean [SD] age, 69.3 [3.8] years; 83 female [61%]), with 2 participants excluded for not meeting eligibility criteria (ie, history of head trauma and amyotrophic lateral sclerosis diagnosis after the 9-month visit [with a likely subclinical state at inclusion]) (Table 1). One non–study-related death (myocardial infarction) was reported at follow-up (data included for estimating intercepts only), and 1 participant did not follow their allocated arm (randomized to no intervention but attended non-native language training) and was analyzed within the non-native language training arm. No between-arm differences in intervention-related metrics were observed (eTable 3 in Supplement 2).

**Table 1.** Sample Baseline Characteristics for Each Trial Arm

Characteristic	Participants, No. (%) (N = 135)		
	Meditation training (n = 45)	Non-native language training (n = 45)	No intervention (n = 45)
Age, mean (SD) [range], y	69.5 (3.7) [65.1-78.4]	70.3 (4.5) [65.0-83.9]	68.1 (2.8) [65.0-76.4]
Sex			
Female	31 (69)	25 (56)	27 (60)
Male	14 (21)	20 (44)	28 (40)
Education, y	13.1 (3.1) [7.0-22.0]	12.2 (3.0) [7.0-17.0]	14.2 (2.9) [7.0-20.0]
APOE ε4 positive	13 (29)	12 (27)	11 (25)^a^
Amyloid deposition SUVR, mean (SD) [range]	1.3 (0.2) [1.0-1.7]^a^	1.3 (0.2) [1.1-1.8]	1.2 (0.1) [1.0-1.5]

Abbreviations: APOE ε4, apolipoprotein ε4 gene variant; SUVR, standard uptake value ratio.

^a^ Forty-four total participants included in this measure.

### Primary Outcomes

**Figure 2.**
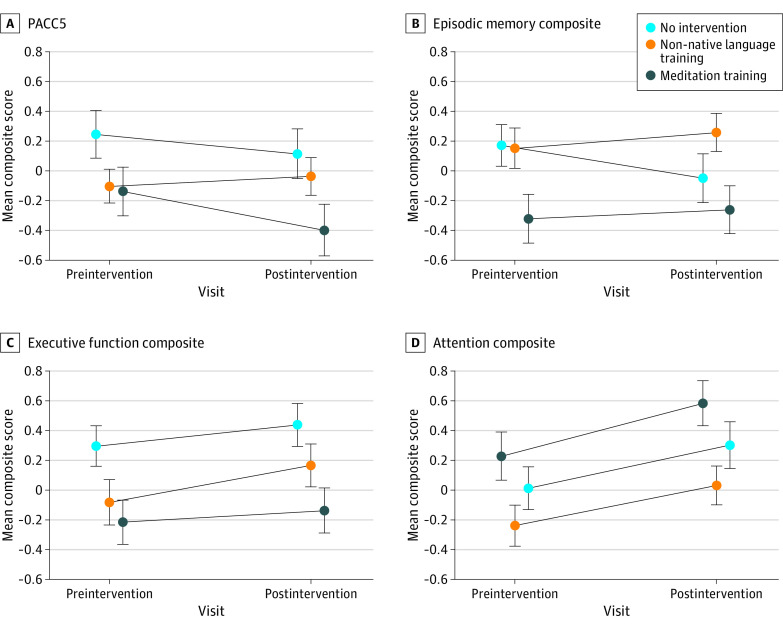
Change in Cognitive Composite Scores From Preintervention to Postintervention Visits by Trial Arm For all composites, higher scores represent better performance. Bars indicate the range of standard error; PACC5, Preclinical Alzheimer Cognitive Composite 5.

The meditation training group showed a decline in PACC5 (standardized estimated change, −0.26; 95% CI, −0.46 to −0.06]) and no improvements in episodic memory (0.06; 95% CI, −0.17 to 0.29) or executive function (0.08; 95% CI, −0.10 to 0.26) (Table 2; Figure 2). The non-native language training group showed no changes in PACC5 (0.07; 95% CI, −0.14 to 0.27) or episodic memory (0.11; 95% CI, −0.13 to 0.34) but exhibited an increase in executive function (0.25; 95% CI, 0.07 to 0.43). The no intervention group did not demonstrate any PACC5 (−0.14; 95% CI, −0.34 to 0.07), episodic memory (−0.23; 95% CI, −0.46 to 0.01), or executive function (0.14; 95% CI, −0.05 to 0.32) changes. All groups showed improvements in attention (meditation training: 0.29; 95% CI, 0.13 to 0.45; non-native language training: 0.27; 95% CI, 0.11 to 0.44; no intervention: 0.35; 95% CI, 0.18 to 0.51) (Table 2 and Table 3; eTable 4 in Supplement 2).

**Table 2.** Descriptive Statistics for the Cognitive Composites by Intervention Group and Visit

Measure	Meditation training				Non-native language training				No intervention control			
	Preintervention		Postintervention		Preintervention		Postintervention		Preintervention		Postintervention	
	No.	Mean (SD)	No.	Mean (SD)	No.	Mean (SD)	No.	Mean (SD)	No.	Mean (SD)	No.	Mean (SD)
PACC5	45	−0.14 (1.10)	45	−0.40 (1.17)	45	−0.10 (0.76)	45	−0.04 (0.85)	45	0.24 (1.07)	44	0.11 (1.12)
Episodic composite	45	−0.32 (1.10)	45	−0.26 (1.08)	45	0.15 (0.91)	45	0.26 (0.86)	45	0.17 (0.94)	44	−0.05 (1.10)
Executive composite	45	−0.21 (1.00)	45	−0.14 (1.01)	45	−0.08 (1.03)	45	0.17 (0.97)	45	0.30 (0.91)	44	0.44 (0.96)
Attention composite	45	0.01 (0.96)	45	0.30 (1.05)	45	−0.24 (0.93)	45	0.03 (0.87)	45	0.23 (1.10)	44	0.58 (1.01)

Abbreviation: PACC5, Preclinical Alzheimer Cognitive Composite 5.

**Table 3.** Model-Adjusted Mean Within-Arm Changes and Between-Arm Differences in Changes for Cognitive Composite Scores^a^

Category	PACC5			Episodic memory			Executive function			Attention		
	Estimate (95% CI)	*P* value	Adjusted *P *value^b^	Estimate (95% CI)	*P* value	Adjusted *P *value^b^	Estimate (95% CI)	*P* value	Adjusted *P *value^b^	Estimate (95% CI)	*P* value	Adjusted *P *value^b^
**Within-arm standardized estimated change**												
Meditation training	−0.26 (−0.46 to −0.06)	NA	NA	0.06 (−0.17 to 0.29)	NA	NA	0.08 (−0.10 to 0.26)	NA	NA	0.29 (0.13 to 0.45)	NA	NA
Non-native language training	0.07 (−0.14 to 0.27)	NA	NA	0.11 (−0.13 to 0.34)	NA	NA	0.25 (0.07 to 0.43)	NA	NA	0.27 (0.11 to 0.44)	NA	NA
No intervention	−0.14 (−0.34 to 0.07)	NA	NA	−0.23 (−0.46 to 0.01)	NA	NA	0.14 (−0.05 to 0.32)	NA	NA	0.35 (0.18 to 0.51)	NA	NA
**Mean difference in change between arms**												
Meditation training vs no intervention	−0.12 (−0.41 to 0.17)	.41	.70	0.29 (−0.04 to 0.61)	.09	.35	−0.06 (−0.32 to 0.20)	.65	.78	−0.06 (−0.29 to 0.17)	.62	.78
Non-native language training vs no intervention	0.20 (−0.08 to 0.49)	.16	.46	0.33 (0.00 to 0.66)	.05	.29	0.12 (−0.15 to 0.37)	.40	.70	−0.08 (−0.31 to 0.16)	.51	.77
Meditation training vs non-native language training	−0.32 (−0.61 to −0.04)	.03	.29	−0.05 (−0.37 to 0.28)	.78	.85	−0.17 (−0.43 to 0.09)	.19	.46	0.02 (−0.21 to 0.25)	.87	.87

Abbreviations: NA, not applicable; PACC5, Preclinical Alzheimer Cognitive Composite 5.

^a^ Adjusted *P* value is for false discovery.

^b^ All analyses were adjusted for age, sex, and education. For within-arm standardized estimated changes, positive values reflect cognitive improvement within a trial arm from preintervention to postintervention; negative coefficients indicate the converse. For the mean difference in change between arms, positive coefficients represent a relatively greater improvement in a trial arm compared with the specified reference trial arm (ie, the trial arm specified after “vs” [either no intervention or non-native language training]); negative coefficients indicate the converse.

No cognitive benefits were found when examining intervention effects through the interaction between visit and trial arm (PACC5: F_2,131.39_ = 2.58; *P* = .08, *P* adjusted for false discovery = .20; episodic memory: F_2,131.60_ = 2.34, *P* = .10, *P* adjusted for false discovery = .20; executive function: F_2,131.26_ = 0.89, *P* = .41, *P* adjusted for false discovery = .55; attention: F_2,131.20_ = 0.24, *P* = .79, *P* adjusted for false discovery = .79).

### Pairwise Comparisons

Meditation training did not show superiority over the no intervention group on any cognitive composite (Table 3). An improvement in episodic memory was observed for non-native language training when compared with the no intervention group; however, the association did not remain following multiple comparison correction (0.33; 95% CI, 0 to 0.66; *P* = .05, *P* adjusted for false discovery = .29). Non-native language training did not confer any beneficial effects on PACC5, executive function, or attention compared with the no intervention group. When comparing the 2 intervention groups on PACC5 changes, non-native language training showed superiority over meditation training, but the association did not survive multiple comparison correction (0.32; 95% CI, 0.04 to 0.61; *P* = .03, *P* adjusted for false discovery = .29). No differences were observed between the intervention groups on other cognitive composites.

### Sensitivity and Exploratory Analyses

Neither the inclusion of amyloid deposition as an additional covariate, exclusion of participants who attended less than 20% of intervention classes nor the exclusion of nonresponders substantively affected results (eTable 5 in Supplement 2). Across both interventions and for all cognitive composites, lower baseline scores were associated with greater improvements. Lower amyloid levels were associated with greater PACC5 and episodic memory gains in the meditation training arm; and with greater executive function improvement but worse episodic memory response in the non-native language training arm. Other characteristics were not consistently associated with intervention response (eTable 6 in Supplement 2).

## Discussion

This study sought to improve scientific understanding of the impacts of meditation training and non-native language training on cognition in cognitively healthy older adults. Contrary to expectations, there was no effect of intervention group on changes in global cognition, episodic memory, executive function, or attention. These findings challenge prevailing hypotheses that meditation training and non-native language training improve cognition in older adults.

Composites sensitive to detecting cognitive changes associated with underlying AD pathology, such as the PACC5, are particularly pertinent for assessing intervention efficacy. We found no evidence for beneficial effects of meditation or non-native language training on PACC5 scores. Despite wide adoption in prospective clinical trials,^24,25^ few RCTs have evaluated nonpharmacological interventions with respect to PACC5 changes.^5^ In the SCD-Well RCT, which included participants with subjective cognitive decline (with lower baseline cognitive scores than Age-Well participants), improvements in PACC5 scores were reported following participation in either an 8-week MBI or health self-management program.^5^ Nonpharmacological interventions may thus confer greater benefits to individuals already experiencing subtle cognitive deficits. Aligning with this hypothesis and earlier studies,^10^ our exploratory analyses revealed that lower baseline cognition was associated with greater improvements; however, it is possible that this pattern reflects what may be described as a regression to the mean.

In regards to episodic memory, there was no evidence of beneficial effects following either intervention. Existing studies provide limited evidence that non-native language training enhances episodic memory.^7^ We observed a small positive effect of non-native language training relative to the no intervention group in pairwise comparisons, although this association did not survive multiple comparison correction. Our results are therefore consistent with extant research indicating that non-native language learning does not elicit cognitive transfer effects on episodic memory.^13,26^ Similarly, there is minimal extant evidence for MBIs having beneficial effects on episodic memory^4^; however, studies comparing long-term meditators with matched controls have reported benefits.^27,28^ Despite Age-Well’s 18-month intervention period, longer interventions may be required to detect cognitive effects in cognitively healthy adults, as MBIs may be more effective in limiting cognitive decline than enhancing cognition.^4^

Theoretical frameworks pose that engagement in meditation confers gains to attention and executive function.^11,29^ With respect to attention, while a positive effect was observed for meditation training, improvements of similar magnitudes were also observed for non-native language training and the no intervention group. Improvements thus likely reflect expectancy or practice effects and align with extant research reporting nondiffering between-group improvements.^4,30^

There were no changes on executive function from preintervention to postintervention. Existing evidence for meditation and non-native language training promoting executive functioning in older adults is mixed. Considering meditation training, 1 meta-analysis found that relative to comparators, MBIs did not improve executive functioning^30^; however, another larger meta-analysis reported positive effects.^4^ In the latter, subdomain analyses (conducted across all age groups, due to insufficient older adult data), revealed improvements in working memory but not inhibition or task switching.^4^ Our executive function composite comprised just 1 (25%) working memory measure, thus any working memory improvements may have been diluted by null effects in other subdomains. Regarding non-native language training, study quality and the executive function skill assessed appear to be determinants of positive effects.^7,9^ For instance, beneficial effects are more frequently observed when working memory and inhibition measures assess accuracy, rather than processing speed.^7,10^ Future trials should therefore investigate intervention effects on executive function subdomains.

Nonpharmacological interventions for the prevention of cognitive decline typically comprise interventions that are distal to the brain (eg, exercise, diet) and/or have limited utility of daily life (eg, cognitive training). Conversely, meditation and non-native language training are more proximal to brain function and the mind and have ecological relevance. Despite this, neither intervention significantly affected Age-Well’s coprimary outcomes (ie, volume and perfusion of the anterior cingulate cortex and insula).^16^ Meditation training, however, positively impacted self-reported attention regulation and socio-emotional capacities.^16^ While these improvements did not translate into improved cognition, they may affect psychoaffective factors associated with dementia risk.

### Strengths and Limitations

Strengths of the present study included its 3-group randomized design and masked administration of a comprehensive cognitive battery. Moreover, both interventions were 18 months in length, making them considerably longer than interventions of comparable content (ie, MBIs: 2 weeks to 13 weeks^4^; non-native language learning: 1 week to 8 months^7^).

The study also had several limitations. Participants, particularly in the no-intervention arm, were highly educated and healthy; this homogeneity limits the generalizability of findings. Furthermore, the interventions may help preserve rather than enhance cognition; as participants were cognitively healthy, effects may therefore only become apparent later with the emergence of cognitive decline. It was also not possible to determine any potential lasting effects of either intervention, as cognition was assessed immediately after interventions ended. However, an ongoing 2-year postintervention visit will allow us to ascertain whether any effects emerge.

## Conclusions

In this secondary analysis of a randomized trial, we investigated the effects of meditation training and non-native language training on 1 global and 3 domain-specific cognitive composites. There was no evidence that either intervention was effective in enhancing cognition. While further analyses are required to explore the effects of meditation and non-native language training on other pertinent aging and well-being outcomes, our findings indicate that the cognitive benefits of these interventions are limited in cognitively healthy older adults.

## Notes

Supplement 1.
Trial Protocol

Supplement 2. **eAppendix 1.** Further Information Relating to Interventions **eAppendix 2.** Further Information Relating to Neuropsychological and Additional Measures **eTable 1.** Age-Well Eligibility Criteria **eTable 2.** Tests Included in the Diagnostic Battery Performed at Screening **eTable 3.** Intervention-Related Metrics **eTable 4.** Model-Adjusted Within-Arm Estimated Changes for Cognitive Composite Scores **eTable 5.** Sensitivity Analyses: Change in Cognitive Composite Scores Compared to the No Intervention Group Following Additional Adjustment for Amyloid Deposition, the Exclusion of Participants Who Did Not Attend at Least 20% of Intervention Classes, and the Exclusion of Nonresponders **eTable 6.** Exploratory Analyses: Investigating Strength of Association Between Candidate Characteristics and Change in Cognitive Composite Scores
eReferences.

Supplement 3.
Nonauthor Collaborators

Supplement 4.
Data Sharing Statement

## References

[1] Panza F, Lozupone M, Logroscino G, Imbimbo BP. A critical appraisal of amyloid-β-targeting therapies for Alzheimer disease. Nat Rev Neurol. 2019;15(2):73-88. doi:10.1038/s41582-018-0116-6 30610216

[2] Synnott PG, Whittington MD, Lin GA, Rind DM, Pearson SD. The effectiveness and value of aducanumab for Alzheimer’s disease. J Manag Care Spec Pharm. 2021;27(11):1613-1617. doi:10.18553/jmcp.2021.27.11.1613 34714106 PMC10391229

[3] Kabat-Zinn J. Mindfulness-based interventions in context: past, present, and future. Clin Psychol Sci Pract. 2003;10:144-156. doi:10.1093/clipsy.bpg016

[4] Whitfield T, Barnhofer T, Acabchuk R, . The effect of mindfulness-based programs on cognitive function in adults: a systematic review and meta-analysis. Neuropsychol Rev. 2022;32(3):677-702. doi:10.1007/s11065-021-09519-y 34350544 PMC9381612

[5] Whitfield T, Demnitz-King H, Schlosser M, ; Medit-Ageing Research Group. Effects of a mindfulness-based versus a health self-management intervention on objective cognitive performance in older adults with subjective cognitive decline (SCD): a secondary analysis of the SCD-Well randomized controlled trial. Alzheimers Res Ther. 2022;14(1):125. doi:10.1186/s13195-022-01057-w 36068621 PMC9446839

[6] Lenze EJ, Voegtle M, Miller JP, . Effects of mindfulness training and exercise on cognitive function in older adults: a randomized clinical trial. JAMA. 2022;328(22):2218-2229. doi:10.1001/jama.2022.21680 36511926 PMC9856438

[7] Ware C, Dautricourt S, Gonneaud J, Chételat G. Does second language learning promote neuroplasticity in aging? a systematic review of cognitive and neuroimaging studies. Front Aging Neurosci. 2021;13:706672. doi:10.3389/fnagi.2021.706672 34867264 PMC8633567

[8] van der Ploeg M, Keijzer M, Lowie W. Methodological concerns and their solutions in third-age language learning studies. Dutch Journal of Applied Linguistics. 2020;9(1-2):97-108. doi:10.1075/dujal.19036.van

[9] Meltzer JA, Kates Rose M, Le AY, . Improvement in executive function for older adults through smartphone apps: a randomized clinical trial comparing language learning and brain training. Neuropsychol Dev Cogn B Aging Neuropsychol Cogn. 2021;30(2):150-171. doi:10.1080/13825585.2021.1991262 34694201

[10] Kliesch M, Pfenninger SE, Wieling M, Stark E, Meyer M. Cognitive benefits of learning additional languages in old adulthood? insights from an intensive longitudinal intervention study. Applied Linguistics. 2022;43(4):653-676. doi:10.1093/applin/amab077

[11] Lutz A, Chételat G, Collette F, Klimecki OM, Marchant NL, Gonneaud J. The protective effect of mindfulness and compassion meditation practices on ageing: hypotheses, models and experimental implementation. Ageing Res Rev. 2021;72:101495. doi:10.1016/j.arr.2021.101495 34718153

[12] Antoniou M, Gunasekera GM, Wong PCM. Foreign language training as cognitive therapy for age-related cognitive decline: a hypothesis for future research. Neurosci Biobehav Rev. 2013;37(10 Pt 2):2689-2698. doi:10.1016/j.neubiorev.2013.09.004 24051310 PMC3890428

[13] Berggren R, Nilsson J, Brehmer Y, Schmiedek F, Lövdén M. Foreign language learning in older age does not improve memory or intelligence: evidence from a randomized controlled study. Psychol Aging. 2020;35(2):212-219. doi:10.1037/pag0000439 32011156

[14] Brown KW, Creswell JD, Ryan RM. Handbook of Mindfulness: Theory, Research, and Practice. Guilford Publications; 2015.

[15] Poisnel G, Arenaza-Urquijo E, Collette F, ; Medit-Ageing Research Group. The Age-Well randomized controlled trial of the Medit-Ageing European project: effect of meditation or foreign language training on brain and mental health in older adults. Alzheimers Dement (N Y). 2018;4:714-723. doi:10.1016/j.trci.2018.10.011 30581977 PMC6296161

[16] Chételat G, Lutz A, Klimecki O, ; Medit-Ageing Research Group. Effect of an 18-month meditation training on regional brain volume and perfusion in older adults: the Age-Well randomized clinical trial. JAMA Neurol. 2022;79(11):1165-1174. doi:10.1001/jamaneurol.2022.3185 36215061 PMC9552046

[17] Zellner Keller B, Singh NN, Winton ASW. Mindfulness-based cognitive approach for seniors (MBCAS): program development and implementation. Mindfulness (N Y). 2014;5(4):453-459. doi:10.1007/s12671-013-0262-2 25067961 PMC4085471

[18] Gilbert P. Compassion-focused therapy: preface and introduction for special section. Br J Clin Psychol. 2014;53(1):1-5. doi:10.1111/bjc.12045 24588759

[19] Rinpoche YM, Swanson E. The Joy of Living: Unlocking the Secret and Science of Happiness. Harmony; 2008.

[20] Schneider LS, Goldberg TE. Composite cognitive and functional measures for early stage Alzheimer’s disease trials. Alzheimers Dement (Amst). 2020;12(1):e12017. doi:10.1002/dad2.12017 32432155 PMC7233425

[21] Papp KV, Rentz DM, Orlovsky I, Sperling RA, Mormino EC. Optimizing the preclinical Alzheimer’s cognitive composite with semantic processing: the PACC5. Alzheimers Dement (N Y). 2017;3(4):668-677. doi:10.1016/j.trci.2017.10.004 29264389 PMC5726754

[22] Devilly GJ, Borkovec TD. Psychometric properties of the credibility/expectancy questionnaire. J Behav Ther Exp Psychiatry. 2000;31(2):73-86. doi:10.1016/S0005-7916(00)00012-4 11132119

[23] Zhang Y, Hedo R, Rivera A, Rull R, Richardson S, Tu XM. Post hoc power analysis: is it an informative and meaningful analysis? Gen Psychiatr. 2019;32(4):e100069. doi:10.1136/gpsych-2019-100069 31552383 PMC6738696

[24] Papp KV, Buckley R, Mormino E, ; Collaborators from the Harvard Aging Brain Study, the Alzheimer’s Disease Neuroimaging Initiative and the Australian Imaging, Biomarker and Lifestyle Study of Aging. Clinical meaningfulness of subtle cognitive decline on longitudinal testing in preclinical AD. Alzheimers Dement. 2020;16(3):552-560. doi:10.1016/j.jalz.2019.09.074 31759879 PMC7067681

[25] Narbutas J, Van Egroo M, Chylinski D, . Associations between cognitive complaints, memory performance, mood, and amyloid-β accumulation in healthy amyloid negative late-midlife individuals. J Alzheimers Dis. 2021;83(1):127-141. doi:10.3233/JAD-210332 34275899

[26] Bubbico G, Chiacchiaretta P, Parenti M, . Effects of second language learning on the plastic aging brain: functional connectivity, cognitive decline, and reorganization. Front Neurosci. 2019;13:423. doi:10.3389/fnins.2019.00423 31156360 PMC6529595

[27] Lykins ELB, Baer RA, Gottlob LR. Performance-based tests of attention and memory in long-term mindfulness meditators and demographically matched nonmeditators. Cognit Ther Res. 2012;36(1):103-114. doi:10.1007/s10608-010-9318-y

[28] Shemesh L, Mendelsohn A, Panitz DY, Berkovich-Ohana A. Enhanced declarative memory in long-term mindfulness practitioners. Psychol Res. 2023;87(1):294-307. doi:10.1007/s00426-022-01642-6 35226153

[29] Vago DR, Silbersweig DA. Self-awareness, self-regulation, and self-transcendence (S-ART): a framework for understanding the neurobiological mechanisms of mindfulness. Front Hum Neurosci. 2012;6:296. doi:10.3389/fnhum.2012.00296 23112770 PMC3480633

[30] Sanchez-Lara E, Lozano-Ruiz A, Perez-Garcia M, Caracuel A. Efficacy of mindfulness-based interventions in cognitive function in the elderly people: a systematic review and meta-analysis. Aging & Mental Health. 2021;26(9):1699-1709. doi:10.1080/13607863.2021.1976724 34587844

