# Supplementary material for: Effects of Meditation Training and Non-Native Language Training on Cognition in Older Adults: A Secondary Analysis of a Randomized Clinical Trial
Source: JAMA Netw Open. 2023 Jul 14;6(7):e2317848. doi: 10.1001/jamanetworkopen.2023.17848 (PMC10349342; doi:10.1001/jamanetworkopen.2023.17848)
Supplement: Supplement 2. — eAppendix 1. Further Information Relating to Interventions eAppendix 2. Further Information Relating to Neuropsychological and Additional Measures eTable 1. Age-Well Eligibility Criteria eTable 2. Tests Included in the Diagnostic Battery Performed at Screening eTable 3. Intervention-Related Metrics eTable 4. Model-Adjusted Within-Arm Estimated Changes for Cognitive Composite Scores eTable 5. Sensitivity Analyses: Change in Cognitive Composite Scores Compared to the No Intervention Group Following Additional Adjustment for Amyloid Deposition, the Exclusion of Participants Who Did Not Attend at Least 20% of Intervention Classes, and the Exclusion of Nonresponders eTable 6. Exploratory Analyses: Investigating Strength of Association Between Candidate Characteristics and Change in Cognitive Composite Scores eReferences. [file jamanetwopen-e2317848-s002.pdf]

Supplemental Online Content

Demnitz-King H, Requier F, Whitfield T, et al; Medit-Ageing Research Group. Effects of meditation training and non-native language training on cognition in older adults: a secondary analysis of a randomized clinical trial. *JAMA Netw Open.* 2023;6(7):e2317848.
doi:10.1001/jamanetworkopen.2023.17848

eAppendix 1. Further Information Relating to Interventions

eAppendix 2. Further Information Relating to Neuropsychological and Additional Measures

eTable 1. Age-Well Eligibility Criteria

eTable 2. Tests Included in the Diagnostic Battery Performed at Screening

eTable 3. Intervention-Related Metrics

eTable 4. Model-Adjusted Within-Arm Estimated Changes for Cognitive Composite Scores

eTable 5. Sensitivity Analyses: Change in Cognitive Composite Scores Compared to the No Intervention Group Following Additional Adjustment for Amyloid Deposition, the Exclusion of Participants Who Did Not Attend at Least 20% of Intervention Classes, and the Exclusion of Nonresponders

eTable 6. Exploratory Analyses: Investigating Strength of Association Between Candidate Characteristics and Change in Cognitive Composite Scores

eReferences.

This supplemental material has been provided by the authors to give readers additional information about their work.

© 2023 Demnitz-King H et al. *JAMA Network Open.*

eTable 1: Age-Well eligibility criteria

**Inclusion criteria**	**Exclusion criteria**
Age ≥ 65 years.	Safety concerns in relation to MR scanning (claustrophobia, ferromagnetic object) or PET scanning (Blood sampling to check hepatic and renal functions are performed before the PET scans; known hypersensibility to Amyvid® or Glucotep®).
Autonomous.	Presence of a major neurological or psychiatric disorder (including an addiction to alcohol or drugs).
Living at home.	History of cerebral disease (vascular, degenerative, physical malformation, tumour, or head trauma with loss of consciousness for more than an hour).
Educational level ≥ 7 years (from the Preparatory Course - 1st grade - included).	Presence of a chronic disease or acute unstable illness (respiratory, cardiovascular, digestive, renal, metabolic, hematologic, endocrine, or infectious).
Registered to the social security system.	Current or recent medication that may interfere with cognitive functioning (psychotropic, antihistaminic with anticholinergic action, anti-Parkinson's, benzodiazepines, steroidal anti-inflammatory long-term treatment, antiepileptic or analgesic drugs), the interfering nature of the different treatments being at the discretion of the investigating doctor.
Motivated to effectively participate in the project and signing the informed consent form.	Being under legal guardianship or incapacitation.
Performance within the normal range on standardized cognitive tests according to agreed study specific standards (age, sex, and education level when available).	Participation in another biomedical research protocol including the injection of radiopharmaceuticals.
Native French speaker.	Physical or behavioural inability to perform the follow-up visits as planned in the study protocol.
Available to attend the intervention for the trial duration (24 months).	
Retired for at least one year.	
No strong preference or aversion for an intervention group.	
No present or past regular or intensive practice of meditation or comparable practices; the practice is considered as regular and/or intensive if it occurred more than one day per week for more than six consecutive months over the last 10 years, and/or in case of more than five consecutive days of intensive practice (internship or retreat) over the past 10 years, and/or of more than 25 days of retreats (cumulatively) within the last 10 years.	
Not speaking fluent English.	

Abbreviations: MR, magnetic resonance; PET, positron emission tomography.

© 2023 Demnitz-King H et al. *JAMA Network Open.*

eTable 2: Tests included in the diagnostic battery performed at screening (V0)

**Domain evaluated**	**Test**	**Score(s)**	**Reference**	**Expected performances**
**Manual laterality**	Edinburgh Questionnaire	Unique	[1]	Not applicable
**Global cognitive functioning**	MMSE	Unique	[2]	Norms according to age, sex, and education level
**Depression**	MADRS	Unique	[3]	Score < 19
**Executive functions**	Wisconsin Card Sorting Test	Multiple	[4]	Z score > −1.65 (norms according to age, sex, and education level)
**Verbal episodic memory**	RL-RI16	Multiple	[5]	Z score > −1.65 (norms according to age, sex, and education level)
**English test**	Evaluation of oral and written comprehension	Unique	Original test	Scores <16/18

Abbreviations: MADRS, Montgomery and Asberg Depression Rating Scale; MMSE, Mini Mental State Examination; RL-RI 16, Rappel libre/Rappel indicé à 16 items.

© 2023 Demnitz-King H et al. *JAMA Network Open.*

eAppendix 1

Interventions
The recruitment of participants was conducted in three waves (divided into three arms) spaced approximately 6 months apart. Each intervention group consisted of between 14 to 17 participants.

Participants assigned to meditation training arm and the non-native language training arm had a standardized intervention program that followed a well-defined plan described in a detailed manual (for the meditation training intervention) or followed a specific program (for the non-native language training intervention) lasting 18 months. Both interventions were delivered by experienced and motivated teachers. The formats of the interventions were identical in terms of the overall course length, class time and home activities, and matched in administration, dosage, duration, and level of expertise and number of facilitators per class.

Accordingly, for both interventions, participants had:

- Group classes of 2 hours, once a week
- Exercises to be done at home every day for at least 20 minutes
- More intensive days and/or half-days of practice

In each weekly group session, there was a time for presentation, a time for sharing, and a time for practice. The first two sessions of each month included an equal amount of these three aspects (3 x 40 minutes), session 3 included more sharing (30/ 60/ 30 minutes), and session 4 included more practice (30/ 30 /60 minutes).

For each intervention, participants were provided with materials (manual and audio) to support their practice. The alternation of supporting material (i.e., texts, images, audio, and video) and activities (i.e., alone, in pairs, and in groups) contributed to maintaining interest and motivation.

Participants were strongly encouraged to participate in all activities over the course of the 18-month intervention period. However, for the duration of the trial participants were asked not to engage in the activities proposed in the arms to which they were not assigned.

Throughout the intervention period participants were asked to fill in a "diary" to record home practice. To facilitate the accurate recording of home practice, an iPad was lent to the participants with all functionalities not required for the trial deactivated (e.g., internet). The tablet contained an application for an iPad 2, which included a questionnaire with two daily questions. Specifically, one question asked participant about the amount of formal practice they engaged in each day (0 minutes, less than 15 minutes, between 15 and 30 minutes, between 30 and 45 minutes, between 45 and 60 minutes, between 60 and 90 minutes, between 90 and 120 minutes, over 120 minutes). Formal practice was defined as practice related to the instructions, exercises, or materials (books and applications) of the course for the non-native language training intervention. For the meditation training group, formal practice was defined as practicing meditation exclusively without doing another activity at the same time. The second question asked participants about the duration of their informal practice (0 minutes, less than 15 minutes, between 15 and 30 minutes, between 30 and 45 minutes, between 45 and 60 minutes, between 60 and 90 minutes, between 90 and 120 minutes, over 120 minutes). In addition to facilitating the reporting of home practice, the iPad allowed participants to access the pedagogical material (audios) used in the interventions.

Meditation training
The meditation intervention for the Medit-Ageing program was delivered by meditation experts and teachers at the Pôle de Formations et de Recherche en Santé (PFRS), in Caen, France.

The original secular meditation training program was offered from a perspective of personal development and successful aging. The 18-month program was intended for healthy retired older adults living at home, with the goal of developing mindfulness and compassion as additional psychological resources for the physical, cognitive, and psychological challenges of aging. The pedagogical content of the meditation intervention was structured into 9 months dedicated to teaching mindfulness meditation followed by 9 months dedicated to teaching caring and compassion meditation. A new teaching theme was introduced each month in the first session and was then explored, practiced, and discussed in the remaining sessions of the month. Each session contained moments of group meditation, sitting or walking, moments of sharing, and moments of teaching.

© 2023 Demnitz-King H et al. *JAMA Network Open.*

Mindfulness is the cultivation of a vigilant awareness of one's own thoughts, actions, emotions, and motivations. The participants were taught to intentionally pay attention to their internal or external experiences in the present moment, without making value judgments. Further, they were encouraged to observe positive (mental calm, compassion) and negative (ruminations, destructive emotions) mental states without identifying with or being absorbed by them. The mindfulness portion of the Medit-Ageing program was directly adapted from an 8-month intervention developed for older adults and validated on a group of French-speaking older adults[6].

The practice of loving-kindness and compassion is aimed at improving the relationship with oneself and the world by addressing emotions such as shame, self-blame, or anger from a more positive perspective, and by developing gratitude and appreciation for positive experiences such as loving-kindness or compassion. The compassion portion of the Medit-Ageing program was directly adapted from Compassion-Focused Therapy (CFT), an integrated and multimodal psychotherapeutic approach developed in England by Prof. Paul Gilbert and adapted in France by Prof. Pascal Delamillieure and Francis Gheysen at the University Hospital of Caen, France[7]. The compassion part of the program incorporated elements from a secular meditation training progam named The Joy of Loving which was developed by the Tibetan Buddhist teacher Mingyur Rinpoche[8]. The adaptation and extension of these two programs for Age-Well were performed by two of the meditation teachers, Martine Batchelor and Thien Huong Tran (Titi Dolma) together with the input from the scientific team.

One day of intensive meditation, that lasted approximately 5 hours, was offered between V2 and V3 (approximately 2 months before the end of the 18-month intervention period).

Non-native language training

The non-native language training (English) intervention was carried out by teachers from the Carré International, a department at the University of Caen dedicated to language learning and who have experience teaching languages to older adults.

A positioning test was administered during the screening visit to evaluate each participant's initial English level and ensure they only had a basic level of English.

When there were greater than 14 participants, sub-groups, based on English proficiency levels, were created to facilitate teaching. Further, starting with the second wave, the non-native language training group was divided into two subgroups to respect logistical constraints on group size. Allocation to each subgroup was drawn randomly via the eCRF randomization module and adapted by the English teachers, if necessary, based on initial English level, to limit the heterogeneity of level within the same group.

In Wave 1, all participants were taught as a single group at a pre-intermediate level (A2-B1 in the Common European Framework of Reference for Languages [CEFRL]). In Wave 2, the participants were split into two groups, both taught at an elementary level (A1-A2 in CEFRL); and in Wave 3, the participants were split into two groups, with one group taught at an elementary level (A1-A2 in CEFRL) and the other at an intermediate level (B2-C1 in CEFRL).

For all groups, the first intervention session was dedicated to creating a group spirit and working on the basics of English. The other sessions consisted of activities to improve oral comprehension and expression, acquisition of new vocabulary, and new grammatical structures. Existing manuals were used, and progress was evaluated using routine methods at Carré International, with personalized or group assistance provided as needed.

One day of intensive English (a day trip to the Anglo-Norman island of Jersey where participants were tasked with obtain information on different places and find objects to buy), that lasted approximately 5 hours, was offered between V2 and V3 (approximately 2 months before the end of the 18-month intervention period).

No intervention

The no intervention group were strongly requested not to change their habits and to continue living as usual. After V3, participants had the opportunity to enrol onto an 8-week mindfulness-based stress reduction intervention (MBSR) or an 8-week English language training program at Caen University.

eAppendix 2

1 Details of individual cognitive measures included in composites

1.1 California Verbal Learning Test II

The California Verbal Learning Test-II (CVLT-II) comprises recalling and recognition of two lists of words over immediate and delayed trials[9]. List A contains 16 words and requires the participant to recall the list over five trials ('sum of trials 1-5'). List B (interference) also contains 16 words and is administered for one trial after List A. Short-delay free recall and cued recall of List A are assessed immediately after the administration of List B. Long-delay recall, long-delay-cued recall, and yes/no-recognition trials of List A follow an approximate delay of 20 minutes. Delayed free recall (range: 0 – 16) is included in the PACC5, whilst the sum of trials 1 – 5 (range: 0 – 80), immediate free recall (range: 0 – 16) and delayed free recall are included in the episodic composite.

1.2 Category fluency

The category fluency test requires participants to generate as many words belonging to the 'animal' category within a two-minute period[10]. The raw total score (i.e., the number of unique eligible responses) was included in the PACC5.

1.3 Mattis Dementia Rating Scale-2

The Mattis Dementia Rating Scale-2 is a standardized scale which assesses a range of cognitive abilities[11]. The scale comprises five subscales: Attention, Initiation/Perseveration, Construction, Conceptualization, and Memory. A total score (range: 0 – 144) is calculated by summing the number of correct performances on each of the subscales. The total score was utilized in the PACC5.

1.4 Digit Span Test

The Digit Span Test is comprised of two trials of eight 'items' and requires participants to repeat a series of numbers (starting with a series of three numbers [e.g., 1-5-9]) in the correct order[12]. Both trials of each item are administered, even if the first trial is answered correctly. An additional number is added to the sequence, until the participant makes a mistake on both trials for a given item. The test can be administered both 'forwards' (i.e., repeating the numbers in the order they are presented) and 'backwards' (i.e., repeating the numbers backwards by starting with the last number and going backward to the first number). For each trial, 1 point is scored for a correct response or 0 points for an incorrect response or no response. The Digit Span forwards score (range 0-16) was included in the attention composite, and the backwards score (range: 0 – 16) was included in the executive function composite.

1.5 Letter fluency

The letter fluency test requires participants to generate as many words beginning with the letter 'P' within a two-minute period[10]. The raw total score (i.e., the number of unique eligible responses) was included in the executive composite.

1.6 Logical Memory Test

During the logical memory test, a short story is orally presented, and the participant is asked to recall the story verbatim (immediate recall; range: 0 – 25)[12]. Approximately 20 minutes later, participants are asked to recall the story verbatim again (delayed recall; range: 0 – 25). Delayed recall scores were included in the PACC5, and the immediate and delayed recall scores in the episodic composite.

1.7 Stroop

The Stroop test comprises three trials, which participants are asked to complete as quickly as possible[10]. In the first, participants are asked to read colour names (printed in black ink; 'word reading') and in the second name different colour swatches (either red, green, or blue; 'colour naming'). In the third trial, colour-words are printed in an inconsistent colour ink (e.g., the word 'red' is printed in blue ink). In this incongruent condition, participants are asked to name the colour of the ink instead of reading the word itself ('colour-word'). The time taken to complete colour-word trial was utilized in the executive composite and the time taken to complete the colour naming trial used included in the attention composite. All scores were multiplied by minus one prior to inclusion in composites, so that higher scores reflected better performance.

1.8 Trail-Making Test

The Trail-Making Test (TMT) is comprised of two parts: TMT-A and TMT-B. In TMT-A participants are required to sequentially connect a series of encircled numbers scattered across the page as fast as possible[10]. In TMT-B, encircled letters are also scattered across the page, in addition to the encircled numbers. Participants

© 2023 Demnitz-King H et al. *JAMA Network Open.*

must sequentially and alternately connect the numbers and letters (e.g., 1 – A – 2 – B, etc). For both conditions, the time to completion (in seconds) is recorded, in addition to the number of errors made. TMT-B completion time was included in the executive composite and TMT-A completion time in the attention composite. All scores were multiplied by minus one prior to inclusion in composites, so that higher scores reflected better performance.

1.9 Wechsler Adult Intelligence Scale (WAIS)-IV Coding
During the WAIS-IV Coding participants are presented with a key in which the numbers 1 – 9 are each paired with a unique geometric symbol[12]. Participants are given 120 seconds to use the key to transcribe the appropriate symbol for a list of numbers between 1 and 9. The number of correctly transcribed geometric symbols are summed to create a total score (range: 0 – 135). WAIS-IV Coding was included in the PACC5 and attention composite.

2 Details of additional measures
2.1 Apolipoprotein E genotyping
Venous blood samples from participants were collected into BD K2-EDTA Vacutainer tubes (BD K2-ethylenediaminetetraacetic acid Vacutainer). APOE genotype was determined following a standardized protocol[13]. Briefly, we used a polymerase chain reaction-based assay that uses HhaI restriction enzymes to digest the PCR products. The resulting digestion fragments were then separated by electrophoresis on polyacrylamide gels. Participants were classified into one of two categories: those with at least one ε4 allele (i.e., APOE ε4+), and those with no ε4 allele (i.e., APOE ε4-).

2.2 Amyloid deposition
All participants were scanned at the Cyceron Center (Caen, France) on the same MRI (Philips Achieva 3.0T scanner) and PET (Discovery RX VCT 64 PET-CT scanner, General Electric Healthcare) cameras.

Structural MRI: A high-resolution T1-weighted anatomical image was acquired using a 3D fast-field echo sequence (3D-T1-FFE sagittal, repetition time =7·1 ms, echo time = 3·3 ms, flip angle = 6°, 180 slices with no gap, slice thickness = 1mm, field of view = 256x256 mm2, in-plane resolution= 1x1x1 mm3). T1-weighted images were segmented using FLAIR images (3D-IR sagittal, TR/TE/TI = 4800/272/1650 ms; flip angle = 40°; 180 slices with no gap; slice thickness = 1 mm; field of view = 250x250 mm2; in-plane resolution = 0·98x0·98 mm2) and spatially normalized to the Montreal Neurological Institute (MNI) template, using the SPM12 segmentation procedure (http://www.fil.ion.ucl.ac.uk). In the present study, MRI scans were used for PET processing only.

PET imaging: Florbetapir-PET scans were acquired with a resolution of 3·76 × 3·76 × 4·9 mm3 (field of view=157mm). Forty-seven planes were obtained with a voxel size of 1·95×1·95×3·27mm3. A transmission scan was performed for attenuation correction before each PET acquisition. Each participant underwent a 10-minute PET scan starting 50 minutes after the intravenous injection of ~4MBq/Kg of Florbetapir. PET images were co-registered on their corresponding anatomical MRI, before being normalized to the MNI template using deformation parameters derived from the anatomical MRI (see above). Resulting images were then scaled using cerebellar grey matter as a reference. Standard uptake value ratios (SUVR) were extracted from specific regions of interest corresponding to AD-sensitive regions, as previously defined. Specifically, normalized and scaled Florbetapir-PET images were used to extract global cortical amyloid SUVR using a predetermined neocortical mask including the entire grey matter, except the following regions: cerebellum, occipital and sensory motor cortices, hippocampi, amygdala and basal nuclei[14].

2.3 Framingham Risk Score
The Framingham Risk score (FRS) is a 7-item index used to quantify an individual's 10-year absolute risk of any cardiovascular disease event occurring[15]. Cardiovascular risk factors included in the risk score are total cholesterol, high-density lipoprotein (HDL) cholesterol, systolic blood pressure, diabetes diagnosis, smoking status, and regular prescription of antihypertensive medication. Items are weighted depending on age and sex and summed. Of note, all participants over the age of 79 years were scored according to the FRS quantified risk for the age group 70-79 years, as the FRS does not include quantified risk for those older than 79 years. Total scores range from -10 to 46, with higher scores indicating a higher 10-year risk of cardiovascular disease.

2.4 Credibility Expectancy Questionnaire
The Credibility Expectancy Questionnaire is a self-report six-item questionnaire aimed at assessing intervention credibility and expectancy for improvement[16]. Three questions form the credibility factor, and three the expectancy factor. Each question is Z-scored based on the distribution of T1 values, then averaged for each

© 2023 Demnitz-King H et al. *JAMA Network Open.*

factor. Scores are then Z-transformed again to create composite scores for credibility and expectancy, with higher scores indicating higher levels of credibility and expectancy.

2.5 Intervention adherence

2.5.1 Session attendance

Attendance at intervention sessions was recorded by intervention facilitators at the start of each session. Interventions were 18-months in length and comprised 2-hour weekly group sessions (ranging between 67 and 71 sessions across recruitment waves) and one day of more intense practice. Attendance at 20% of sessions (i.e., 14 sessions) was the a priori determined adequate minimum dose[17]. Of note, although participants in wave 2 of the meditation training arm were offered a total of 72 sessions, thus making 15 sessions the cut-off, using a 15-session cut-off does not change any results (i.e., no additional participants would fall below the cut off).

2.5.2 Total practice

The extent to which participants engaged with their assigned intervention was calculated by combining the number of minutes spent in class attendance actively engaging in intervention activities (i.e., 60 minutes per session and 5 hours for the intensive day) with the number of minutes participants reported engaging in formal practices outside of classes. Participants in both intervention groups were provided with a homework diary and asked to record the amount of time they spent engaging with formal practices each day for the duration of the trial.

2.6 Intervention 'responders' and 'non-responders'

Across both intervention groups, whether, and the degree to which participants responded to their assigned intervention was assessed. Specifically, a dichotomous variable classifying participants as either intervention 'responders' or 'non-responders', and a continuous measure of responsiveness were computed.

2.6.1 Categorical approach

For the meditation training group, data gathered from facilitators at the end of the 18-month intervention period was used to classify participants as either 'responders' or 'non-responders'. Specifically, facilitators were jointly asked to rate the extent to which they believed each participant benefited from the intervention on a Likert scale which ranged from 0 ('pas du tout significative' [translation: 'not significantly at all']) to 5 ('énormément' [translation: 'very much']). Participants who received a score of 0 or 1 were classified as 'non-responders'.

For the non-native language training group, participants performance on an English language test and facilitator ratings were utilized to classify participants as either 'responders' or 'non-responders'. Specifically, a participant was considered a 'non-responder' if they received an average facilitator rating score of 0 or 1, and their improvement on an English language test (i.e., delta score created by subtracting V1 scores from V3 scores) was less than 1 point.

2.6.2 Continuous approach

For the meditation training group, a continuous measure of responsiveness was computed by combining standardized scores from three domains: (i) change from V1 to V3 on a global meditation composite score; (ii) facilitator ratings of participants' response to the intervention; and (iii) participants' perceived response to the intervention. A total of 15 (sub)scales from six self-report measures were utilized to create the global meditation composite score at V1 and V3. Details pertaining to the scales included in the composite and the methods used to create it have previously been described [18]. Facilitator ratings of participants' response to the intervention were assessed via the previously described question assessing perceived benefit (see 'Categorical approach' sub-section) and facilitator responses to Scale B. Scale B captured facilitators' perception of participants' general levels of connection, positive emotions, negative emotions, and meta-awareness. Participant ratings on Scale A and Scale B were utilized to capture participants' perceived response to the intervention. Scale A captured how beneficial participants perceived the intervention to be during the sessions, whilst Scale B captured the extent to which participants perceived the intervention to have a beneficial effect on their daily lives. To create the continuous measure of responsiveness for participants in the meditation training arm, all five sub-scores were first standardized using relevant means and standard deviations. The sub-scores within the second (i.e., facilitator ratings) and third (i.e., participant self-ratings) domain were averaged, and then re-standardized to yield two scores. Lastly, the three standardized domain scores were averaged and the final responsiveness variable re-standardized to have a mean of 0 and a standard deviation of 1.

For the non-native language training group, a continuous measure of responsiveness was computed by combining standardized scores from two domains: (i) change from V1 to V3 on an English test; and (ii) teacher ratings of participants' response to the intervention. Both constituents have previously been described (see

© 2023 Demnitz-King H et al. *JAMA Network Open.*

‘Categorical approach’ sub-section). To create a continuous measure of responsiveness for participants in the non-native language training arm, both sub-scores were first standardized using the relevant means and standard deviations. The two standardized domain scores were then averaged and re-standardized create the final responsiveness variable, with a mean of 0 and standard deviation of 1.

© 2023 Demnitz-King H et al. *JAMA Network Open.*

eTable 3. Intervention-related metrics

	**Meditation training** (*n* = 45)	**Non-native language training** (*n*= 45)	**Difference between intervention groups**
CEQ-credibility	-0.1 (1·0) [-2.5 - 1.1]	0·1 (1·0) [-3.6 - 1.1]	*P* = 0·300 *
CEQ-expectancy	0·1 (0·9) [-1·9 - 1·7]	-0.1 (1·1) [-3.2 - 1·7]	*P* = 0·339 *
Session attendance	60·6 (8·2) [26·0 - 70·0]	58·1 (12·3) [7·0 - 68·0]	*P* = 0·254
Practice, *minutes*	17971·3 (9633·3) [1762·5 - 40012·5]	18072·6 (8077·6) [1492·5 – 46170·0]	*P* = 0·789
Responder, *n* (%)	38 (84·4%)	43 (95·6%)	*P* = 0·157 †

Note: Data are presented as means (SD) [range], unless otherwise stated. *Superscripts:* * *P*-value from Mann-Whitney U test reported; † *P*-value from Fisher's exact test reported. *Abbreviations:* CEQ, Credibility/Expectancy Questionnaire.

© 2023 Demnitz-King H et al. *JAMA Network Open.*

eTable 4. Model-Adjusted Within-Arm Estimated Changes for Cognitive Composite Scores

Category	PACC5, estimate (95% CI)	Episodic memory, estimate (95% CI)	Executive function, estimate (95% CI)	Attention, estimate (95% CI)
Meditation training	−0.26 (−0.46 to −0.06)	0.06 (−0.17 to 0.29)	0.08 (−0.10 to 0.26)	0.29 (0.13 to 0.45)
Non-native language training	0.07 (−0.14 to 0.27)	0.11 (−0.13 to 0.34)	0.25 (0.07 to 0.43)	0.27 (0.11 to 0.44)
No intervention	−0.14 (−0.34 to 0.07)	−0.23 (−0.46 to 0.01)	0.14 (−0.05 to 0.32)	0.35 (0.18 to 0.51)

© 2023 Demnitz-King H et al. *JAMA Network Open.*

eTable 5. Sensitivity analyses: Change in cognitive composite scores compared to the no intervention group following: (i) additional adjustment for amyloid deposition; (ii) the exclusion of participants who did not attend at least 20% of intervention classes and (iii) the exclusion of 'non-responders'.

		**Linear mixed model analyses with interaction**								
		**(i) Amyloid deposition**			**(ii) Session attendance**			**(iii) 'Responder'**		
		**Omnibus**								
**PACC5**	Visit * Group	F(2, 130.40)=3.03, *P*=0.05, P_{FDR}=0.20			F(2, 130.39)=2.64, *P*=0.08, P_{FDR}=0.16			F(2, 122.35)=1.89, P=0.16, P_{FDR}=0.31		
**Episodic memory**	Visit * Group	F(2, 130.61)=2.36, *P*=0.10, P_{FDR}=0.20			F(2, 130.60)=2.56, *P*=0.08, P_{FDR}=0.16			F(2, 122.54)=2.48, P=0.09, P_{FDR}=0.31		
**Executive function**	Visit * Group	F(2, 130.31)=1.11, *P*=0.33, P_{FDR}=0.44			F(2, 130.26)=0.94, *P*=0.39, P_{FDR}=0.52			F(2, 122.25)=1.05, P=0.34, P_{FDR}=0.34		
**Attention**	Visit * Group	F(2, 130.23)=0.22, *P*=0.80, P_{FDR}=0.80			F(2, 130.23)=0.25, *P*=0.78, P_{FDR}=0.78			F(2, 122.18)=0.37, P=0. 34, P_{FDR}=0.34		
		**Pairwise comparisons**								
		Estimate (CI)	*P*	P_{FDR}	Estimate (CI)	*P*	P_{FDR}	Estimate (CI)	*P*	P_{FDR}
**PACC5**	Meditation training vs. No intervention	-0·15 (-0·43, 0·13)	0·30	0.60	-0·12 (-0·40, 0·16)	0·41	0.70	-0·07 (-0·37, 0·22)	0·63	0.84
	Non-native language training vs. No intervention	0·20 (-0·08, 0·48)	0·16	0.38	0·21 (-0·08, 0·50)	0·15	0.43	0·21 (-0·08, 0·50)	0·16	0.44
	Meditation training vs. Non-native language training	-0.35 (-0.63, -0.07)	0.02	0.24	-0.33 (-0.62, -0.05)	0.02	0.24	-0.28 (-0.58, 0.02)	0.07	0.28
**Episodic memory**	Meditation training vs. No intervention	0·29 (-0·03, 0·62)	0·08	0.32	0·29 (-0·04, 0·61)	0·09	0.36	0·33 (0·01, 0·66)	0·05	0.28
	Non-native language training vs. No intervention	0·33 (0·01, 0·65)	0·05	0.30	0·35 (0·03, 0·68)	0·04	0.24	0·30 (-0·02, 0·62)	0·07	0.28
	Meditation training vs. Non-native language training	-0.04 (-0.36, 0.29)	0.83	0.83	-0.07 (-0.39, 0.25)	0.68	0.74	0.03 (-0.29, 0.36)	0.84	0.92
**Executive function**	Meditation training vs. No intervention	-0·08 (-0·34, 0·17)	0·53	0.71	-0·06 (-0·32, 0·19)	0·65	0.74	-0·00 (-0·26, 0·25)	0·99	0.99
	Non-native language training vs. No intervention	0·11 (-0·14, 0·36)	0·39	0.67	0·11 (-0·14, 0·37)	0·37	0.70	0·16 (-0·09, 0·41)	0·21	0.44
	Meditation training vs. Non-native language training	-0.19 (-0.44, 0.06)	0.14	0.38	-0.18 (-0.43, 0.08)	0.18	0.43	-0.16 (-0.42, 0.09)	0.22	0.44
**Attention**	Meditation training vs. No intervention	-0·05 (-0·28, 0·18)	0·67	0.80	-0·06 (-0·29, 0·17)	0·62	0.74	-0·10 (-0·34, 0·13)	0·40	0.69
	Non-native language training vs. No intervention	-0·08 (-0.31, 0.15)	0·51	0.71	-0·08 (-0·32, 0·15)	0·49	0.74	-0·07 (-0·29, 0·16)	0·58	0.84
	Meditation training vs. Non-native language training	0.03 (-0.20, 0.26)	0.82	0.83	0.02 (-0.21, 0.25)	0.85	0.85	-0.04 (-0.27, 0.20)	0.76	0.91

Note: The regression coefficients reported here are standardized. All linear mixed models were adjusted for age, sex, years of education. The 'Amyloid deposition' sensitivity analyses additionally adjust for global amyloid SUVRs; the 'Session attendance' sensitivity analyses excluded participants who did not attend at least 20% of intervention classes; and the 'Responder' sensitivity analyses excluded participants who were classified as 'non-responder'. *Abbreviations: CI*, Confidence interval; *FDR*, false discovery rate; PACC5, Preclinical Alzheimer Cognitive Composite 5; *SUVR*, Standard uptake value ratios.

© 2023 Demnitz-King H et al. *JAMA Network Open.*

eTable 6. Exploratory analyses: Investigating strength of association between candidate characteristics and change in cognitive composite scores.

Variables of Interest	Meditation training				Foreign language training			
	**PACC5**	**Episodic composite**	**Executive composite**	**Attention composite**	**PACC5**	**Episodic composite**	**Executive composite**	**Attention composite**
	Estimate (95% CI)							
Age	-0·01 (-0·06, 0·03)	0·01 (-0·04, 0·06)	0·00 (-0·04, 0·04)	-0·03 (-0·06, 0·01)	-0·02 (-0·04, 0·01)	0·03 (-0·01, 0·06)	-0·00 (-0·03, 0·02)	-0·00 (-0·03, 0·03)
Sex [a]	-0·10 (-0·44, 0·24)	0·19 (-0·19, 0·57)	0·06 (-0·26, 0·39)	0·02 (-0·24, 0·29)	0·22 (-0·03, 0·48)	0·32 (-0·00, 0·63)	0·21 (-0·07, 0·48)	**-0·34 (-0·61, -0·07)**
Education	-0·03 (-0·08, 0·02)	-0·00 (-0·06, 0·06)	-0·00 (-0·05, 0·05)	0·01 (-0·03, 0·05)	**0·05 (0·01, 0·09)**	0·01 (-0·04, 0·07)	0·02 (-0·03, 0·07)	0·01 (-0·04, 0·05)
APOE [b]	0·04 (-0·31, 0·40)	0·15 (-0·23, 0·54)	0·06 (-0·27, 0·40)	0·27 (-0·00, 0·53)	**-0·27 (-0·54, -0·01)**	-0·29 (-0·63, 0·05)	**0·39 (0·10, 0·69)**	-0·08 (-0·38, 0·22)
Amyloid deposition	**-0·24 (-0·39, -0·09)**	**-0·21 (-0·39, -0·04)**	-0·01 (-0·16, 0·13)	-0·05 (-0·17, 0·08)	0·12 (-0·00, 0·25)	**0·24 (0·09, 0·40)**	**-0·21 (-0·34, -0·07)**	-0·00 (-0·14, 0·14)
Credibility	0·10 (-0·07, 0·28)	-0·00 (-0·20, 0·19)	**-0·22 (-0·38, -0·06)**	0·05 (-0·08, 0·19)	-0·07 (-0·20, 0·06)	**-0·21 (-0·38, -0·05)**	0·11 (-0·04, 0·26)	0·06 (-0·09, 0·20)
Expectancy	0·14 (-0·02, 0·31)	0·05 (-0·14, 0·23)	-0·15 (-0·31, 0·00)	-0·01 (-0·14, 0·12)	-0·05 (-0·18, 0·08)	**-0·20 (-0·36, -0·04)**	0·00 (-0·14, 0·15)	0·08 (-0·06, 0·22)
Wave [c]								
Wave 2	-0·31 (-0·70, 0·08)	**-0·60 (-1·01, -0·18)**	**-0·36 (-0·71, -0·01)**	**0·32 (0·03, 0·62)**	-0·13 (-0·45, 0·20)	-0·21 (-0·63, 0·20)	0·29 (-0·07, 0·64)	0·07 (-0·29, 0·43)
Wave 3	0·07 (-0·33, 0·46)	-0·26 (-0·68, 0·16)	0·21 (-0·15, 0·57)	-0·01 (-0·31, 0·29)	-0·29 (-0·62, 0·03)	-0·04 (-0·46, 0·38)	0·03 (-0·33, 0·39)	0·17 (-0·19, 0·53)
Baseline score	**-0·23 (-0·41, -0·05)**	**-0·32 (-0·48, -0·16)**	**-0·29 (-0·46, -0·13)**	**-0·17 (-0·31, -0·03)**	**-0·25 (-0·44, -0·07)**	**-0·41 (-0·61, -0·21)**	**-0·32 (-0·46, -0·18)**	**-0·35 (-0·48, -0·21)**
Total practice	0·08 (-0·08, 0·25)	0·09 (-0·09, 0·28)	-0·10 (-0·25, 0·06)	-0·07 (-0·20, 0·06)	-0·01 (-0·14, 0·12)	0·01 (-0·14, 0·16)	0·06 (-0·07, 0·19)	0·03 (-0·11, 0·17)
FRS	-0·08 (-0·24, 0·09)	-0·06 (-0·23, 0·12)	0·05 (-0·11, 0·20)	-0·03 (-0·16, 0·10)	-0·02 (-0·14, 0·10)	-0·12 (-0·28, 0·03)	-0·09 (-0·23, 0·04)	**0·23 (0·11, 0·36)**
Responsiveness	0·10 (-0·07, 0·27)	0·08 (-0·11, 0·27)	0·01 (-0·15, 0·17)	-0·11 (-0·24, 0·02)	0·02 (-0·11, 0·15)	-0·10 (-0·26, 0·06)	0·12 (-0·02, 0·26)	0·05 (-0·09, 0·19)

Note: The regression coefficients reported here are standardized. All analyses were adjusted for age, sex, and years of education, except for FRS analyses where only education was included as covariate because the FRS includes age and sex in its scoring. **Emboldened** coefficient estimates and 95% CIs had *p*-values <0.05. *Superscripts:* [a]Reference sex is male. [b]Reference APOE genotype is no ε4 allele. [c]Reference wave is wave 1. *Abbreviations: APOE*, Apolipoprotein E; *CI*, Confidence interval; *FRS,* Framingham Risk Score; *PACC5*, Preclinical Alzheimer Cognitive Composite 5; *SUVR*, Standard uptake value ratio.

© 2023 Demnitz-King H et al. *JAMA Network Open.*

eReferences

1 Oldfield RC. The assessment and analysis of handedness: the Edinburgh inventory. *Neuropsychologia* 1971; **9**: 97–113.

2 Folstein MF, Folstein SE, McHugh PR. “Mini-mental state”: a practical method for grading the cognitive state of patients for the clinician. *Journal of psychiatric research* 1975; **12**: 189–98.

3 Montgomery SA, Åsberg M. A new depression scale designed to be sensitive to change. *The British journal of psychiatry* 1979; **134**: 382–9.

4 Grant DA, Berg E. A behavioral analysis of degree of reinforcement and ease of shifting to new responses in a Weigl-type card-sorting problem. *Journal of experimental psychology* 1948; **38**: 404.

5 Van der Linden M, Adam S, Agniel A, Baisset Mouly C. L’évaluation des troubles de la mémoire: Présentation de quatre tests de mémoire épisodique (avec leur étalonnage). *Neuropsychologie* 2004.

6 Zellner Keller B, Singh NN, Winton ASW. Mindfulness-Based Cognitive Approach for Seniors (MBCAS): Program Development and Implementation. *Mindfulness (N Y)* 2014; **5**: 453–9.

7 Gilbert P. Compassion-focused therapy: Preface and introduction for special section. *British Journal of Clinical Psychology* 2014; **53**: 1–5.

8 Rinpoche YM, Swanson E. The joy of living: Unlocking the secret and science of happiness. Harmony, 2008.

9 Poitrenaud J, Deweer B, Kalafat M, Van der Linden M. Adaptation en langue française du California Verbal Learning Test. *Paris: Les Éditions du Centre de psychologie appliquée* 2007.

10 Godefroy Olivier, Groupe de réflexion pour l’évaluation des fonctions exécutives, Godefroy Olivier. Fonctions exécutives et pathologies neurologiques et psychiatriques : évaluation en pratique clinique / coordonné par Godefroy O. et le Groupe de réflexion pour l’évaluation des fonctions exécutives. Marseille: Solal, 2008.

11 Groupe de réflexion sur les évaluations cognitives (GRECO). Échelle d’évaluation de la démence de Mattis. Manuel de passation. Version française consensuelle. Retrieved from http://site-greco.net/ download/ MATTIS_FeuillesEx.pdf, 1994.

12 Wechsler D. Wechsler Adult Intelligence Scale--Fourth Edition. 2008. DOI:10.1037/t15169-000.

13 Rousseaux S., Guerber F., Valenti K., Hadjian A-J. Le polymorphisme de l’apolipoprotéine E : intérêt, détermination simple par PCR. *Revue française des laboratoires* 1992; **n°244**.

14 Besson FL, La Joie R, Doeuvre L, *et al.* Cognitive and Brain Profiles Associated with Current Neuroimaging Biomarkers of Preclinical Alzheimer’s Disease. *J Neurosci* 2015; **35**: 10402–11.

15 D’Agostino RB, Vasan RS, Pencina MJ, *et al.* General Cardiovascular Risk Profile for Use in Primary Care. *Circulation* 2008; **117**: 743–53.

16 Devilly GJ, Borkovec TD. Psychometric properties of the credibility/expectancy questionnaire. *Journal of Behavior Therapy and Experimental Psychiatry* 2000; **31**: 73–86.

© 2023 Demnitz-King H et al. *JAMA Network Open.*

17 Poisnel G, Arenaza-Urquijo E, Collette F, *et al.* The Age-Well randomized controlled trial of the Medit-Ageing European project: Effect of meditation or foreign language training on brain and mental health in older adults. *Alzheimers Dement (N Y)* 2018; **4**: 714–23.

18 Schlosser M, Barnhofer T, Requier F, *et al.* Measuring Psychological Mechanisms in Meditation Practice: Using a Phenomenologically Grounded Classification System to Develop Theory-Based Composite Scores. *Mindfulness* 2022; published online Jan 31. DOI:10.1007/s12671-021-01816-0.

© 2023 Demnitz-King H et al. *JAMA Network Open.*
