# Supplementary material for: Effects of Meditation Training and Non-Native Language Training on Cognition in Older Adults: A Secondary Analysis of a Randomized Clinical Trial
Source: JAMA Netw Open. 2023 Jul 14;6(7):e2317848. doi: 10.1001/jamanetworkopen.2023.17848 (PMC10349342; doi:10.1001/jamanetworkopen.2023.17848)
Supplement: Supplement 3. — Nonauthor Collaborators [file jamanetwopen-e2317848-s003.pdf]

Supplemental Online Content: Nonauthor Collaborators
*First name, last name, and suffix (if applicable) are required and will appear in PubMed.

*Group Name(s): Medit-Ageing Research Group							
***First Name and Middle Initial(s)**	***Last Name**	***Suffix (eg, Jr, III)**	Academic Degrees	Institution	Location (city, state/province, country)	Role or Contribution, eg, chair, principal investigator	Group (if more than 1 Group listed in the byline) and/or Subgroup (eg, Steering Committee)
Florence	Allais		BSc	INSERM	Bordeaux, France	Data manager	
Claire	André		PhD	INSERM	Caen, France	PhD student	
Eider	Arenaza-Urquijo		PhD	INSERM	Caen, France	Post-doctoral position	
Julien	Asselineau		MSc	INSERM	Bordeaux, France	Statistician	
Sebastian	Baez Lugo		PhD	UNIGE	Geneva, Switzerland	PhD student	
Martine	Batchelor			Independent	Bordeaux, France	Meditation teacher	
Axel	Beaugonin			Independent	Caen/Paris, France	Meditation teacher	
Alexandre	Bejanin		PhD	INSERM	Caen, France	Post-doctoral position	
Maelle	Botton		MSc	INSERM	Caen, France	Neuropsychologist	
Pierre	Champetier		MSc	UNICAEN	Caen, France	PhD student	
Anne	Chocat		MD	INSERM	Caen, France	Investigating medical doctor	
Robin	De Flores		PhD	INSERM	Caen, France	Post-doctoral position	
Vincent	De La Sayette		MD PHD	CHU Caen	Caen, France	Principle investigating medical doctor	
Pascal	Delamilleure		MD PhD	CHU Caen	Caen, France	Meditation teacher	
Stéphanie	Egret		MSc	INSERM	Caen, France	Neuropsychologist	
Hélene	Espérou		MD	INSERM	Paris, France	Sponsor	
Francesca	Felisatti		MSc	INSERM	Caen, France	PhD student	
Eglantine	Ferrand-Devouges		MD	INSERM	Bordeaux, France	Methodologist	
Antoine	Garnier-Groussard		MD MSc	CHU Lyon	Lyon, France	PhD student	
Francis	Gheysen		MD	Independent	Caen, France	Meditation teacher	
Marc	Heidmann		MSc	INSERM	Lyon, France	PhD student	

Supplemental Online Content: Nonauthor Collaborators
*First name, last name, and suffix (if applicable) are required and will appear in PubMed.

***First Name and Middle Initial(s)**	***Last Name**	***Suffix (eg, Jr, III)**	Academic Degrees	Institution	Location (city, state/province, country)	Role or Contribution, eg, chair, principal investigator	Group (if more than 1 Group listed in the byline) and/or Subgroup (eg, Steering Committee)
Anne	Hendy		MSc	UCL	London, UK	MSc student	
Thien	Huong Tran			INSERM	Paris, France	Meditation teacher	
Agathe	Joret Philippe		MSc	INSERM	Caen, France	Neuropsychologist	
Elizabeth	Kuhn		PhD	INSERM	Caen, France	PhD student	
Brigitte	Landeau		MSc	INSERM	Caen, France	Neuroimaging development engineer	
Gwendoline	Le Du		MSc	INSERM	Caen, France	Technician	
Valérie	Lefranc		BA	INSERM	Caen, France	Technician	
Florence	Mezenge		BA	INSERM	Caen, France	Neuroimaging engineer assistant	
Inés	Moulinet		PhD	INSERM	Caen, France	PhD student	
Valentin	Ourry		PhD	INSERM	Caen, France	PhD student	
Cassandre	Palix		MSc	INSERM	Caen, France	PhD student	
Anne	Quillard		MD	INSERM	Caen, France	Researcher	
Géraldine	Rauchs		PhD	INSERM	Caen, France	Researcher	
Stéphane	Rehel		PhD	INSERM	Caen, France	PhD student	
Corrine	Schwimmer		MSc	UNICAEN	Caen, France	English teacher	
Siya	Sherif		PhD	INSERM	Caen, France	Research engineer	
Clémence	Tomadesso		PhD	INSERM	Caen, France	PhD student	
Edelweiss	Touron		PhD	INSERM	Caen, France	PhD student	
Matthieu	Vanhoutte		PhD	INSERM	Caen, France	Post-doctoral position	
