# Supplementary material for: Effects of Meditation Training and Non-Native Language Training on Cognition in Older Adults: A Secondary Analysis of a Randomized Clinical Trial
Source: JAMA Netw Open. 2023 Jul 14;6(7):e2317848. doi: 10.1001/jamanetworkopen.2023.17848 (PMC10349342; doi:10.1001/jamanetworkopen.2023.17848)
Supplement: Supplement 4. — Data Sharing Statement [file jamanetwopen-e2317848-s004.pdf]

Data Sharing Statement

Demnitz-King. Effects of Meditation Training and Non-native Language Training on Cognition in Older Adults. *JAMA Netw Open*. Published July 14, 2023.
doi:10.1001/jamanetworkopen.2023.17848

Data
Data available: Yes
Data types: Deidentified participant data, Data dictionary
How to access data: The study protocol, including the statistical analysis plan, is available online at: 10.1016/j.trci.2018.10.011. The datasets used and/or analysed during the current study are available from the corresponding author on reasonable request, subject to approval by the project executive committee and study sponsor. To gain access, researchers will need to submit a data request form.
When available: With publication

Supporting Documents
Document types: None

Additional Information
Who can access the data: NA
Types of analyses: NA
Mechanisms of data availability: NA
Any additional restrictions: NA
